# Prognostic value of preoperative peripheral blood mean platelet volume/platelet count ratio (MPV/PC) in patients with resectable cervical cancer

**DOI:** 10.1186/s12885-021-09016-8

**Published:** 2021-11-29

**Authors:** Qicheng Deng, Qifang Long, Yanan Liu, Zhujuan Yang, Yibei Du, Xin Chen

**Affiliations:** Department of Obstetrics and Gynecology, The Second Affiliated Hospital of Soochow University, 1055 Sanxiang Street, Suzhou, 215004 Jiangsu Province China

**Keywords:** MPV/PC, Cervical cancer, Prognosis, Nomogram

## Abstract

**Background:**

The mean platelet volume/platelet count ratio (MPV/PC) ratio based on the preoperative peripheral MPV and PCcan be used to predict the prognosis of multiple malignant tumors.

**Objective:**

To evaluate the prognostic value of MPV/PC in cervical cancer patients.

**Methods:**

This study enrolled 408 patients who had undergone radical surgery for cervical cancer and evaluated the correlation of MPV/PC with patient prognosis in the primary cohort and validation cohort. Additionally, independent prognostic factors were incorporated to construct the prognostic nomogram, and the area under the receiver operating characteristic (ROC) curve (AUC) value was calculated to analyze the prognostic predictive ability of the nomogram.

**Results:**

In the primary cohort, Kaplan–Meier survival analysis indicated that the overall survival (OS) for patients with MPV/PC ≤ 0.41 was significantly lower than that in patients with MPV/PC > 0.41. MPV/PC was an independent prognostic factor for resectable cervical cancer patients. Compared with neutrophil/lymphocyte ratio (NLR), platelet/lymphocyte ratio (PLR) or monocyte/lymphocyte ratio (MLR), the AUC values of MPV/PC in predicting the 3- and 5-year survival rates for cervical cancer patients were greater. Similar results were verified in the validation cohort. Subsequently, the nomogram constructed based on MPV/PC, International Federation of Gynecology and Obstetrics (FIGO) classification and lymphovascular invasion performed well to accurately predict the prognosis of cervical cancer patients. The 3- and 5-year survival rates predicted by the nomogram were highly consistent with the real observations. Similar results were also displayed in the validation cohort.

**Conclusions:**

MPV/PC may be used as a novel independent prognostic factor for patients with resectable cervical cancer. Compared with the FIGO classification system, the nomogram integrating MPV/PC maybe reliably predict the survival of cervical cancer patients after radical surgery.

## Introduction

The morbidity and mortality of cervical cancer rank 4th among female malignant tumors worldwide [1]. Globally, approximately 527,600 new cervical cancer cases and 265,700 cervical cancer-related deaths are reported annually [1]; among them, more than 80% of cases come from developing countries. According to statistics, 98,900 new cervical cancer cases and 30,500 deaths occurred in China in 2015 [2]. Currently, the International Federation of Gynecology and Obstetrics (FIGO) classification system is mainly used to judge the clinical prognosis of cervical cancer. However, the FIGO classification system is restricted in judging the prognosis of cervical cancer patients. So, other indexes must be applied (such as hematological indexes, pathological type, histological grade, tumor infiltration depth or scope, and lymph node metastasis (LNM) staging) to comprehensively judge the patient prognosis.

In recent years, hematological indexes have attracted extensive attention regarding their predictive value in malignant tumors [3, 4]. In cervical cancer, the neutrophil/lymphocyte ratio (NLR), platelet/lymphocyte ratio (PLR), monocyte/lymphocyte ratio (MLR) and systemic inflammation response index (SIRI) are closely related to the prognosis of cervical cancer patients [5]. Recent studies have indicated that the MPV/PC ratio based on the mean platelet volume (MPV) and platelet count (PC) can predict the prognosis of multiple malignant tumors [6–12]. However, the prognostic value of MPV/PC in cervical cancer has not yet been investigated. Therefore, this study aimed to explore the influencing factors for the prognosis of cervical cancer patients, evaluate the prognostic values of MPV/PC, NLR, PLR and MLR in cervical cancer, and construct a prognostic nomogram for resectable cervical cancer patients on this basis. Additionally, the prognostic prediction accuracy of this model was compared with that of the FIGO classification system to guide clinical practice and improve the clinical outcomes of cervical cancer patients.

## Materials and methods

### Clinical data of patients

Together, 283 patients who received radical surgery for cervical cancer at the Second Affiliated Hospital of Soochow University from 2009 to 2017 were retrospectively analyzed. The inclusion criteria were as follows: 1. patients pathologically diagnosed with primary cervical squamous carcinoma with FIGO stage IA-IIA; 2. patients with no chronic heart, liver, kidney disease, diabetes, or severe infection; 3. patients who did not take antiplatelet agents or receive anticoagulation therapy within 1 month before examination; 4. patients with complete clinical and follow-up data; and 5. patients who did not receive neoadjuvant chemotherapy (NCT) or radiotherapy. The follow-up plan after initial treatment comprised re-examinations every 3 months within 1–2 years after surgery, every 6 months within 3–5 years and yearly thereafter. The follow-up examinations mainly included interrogation, gynecological examination, cervical cytological examination, transvaginal ultrasonography, and CT or MRI. Overall survival (OS) was defined as the time from the date of surgery to the date of death from any cause or the last follow-up for the survivors or those lost to follow-up (censor). The last follow-up was conducted on June 30th, 2019, and the median follow-up period was 72 (range, 4–129) months. Additionally, 125 cervical cancer patients who had undergone radical resection at the Kunshan First People’s Hospital were recruited as the validation cohort. The study design conformed to the Declaration of Helsinki and was approved by the Ethics Committee of the Second Affiliated Hospital of Soochow University (2009-KY-043). All the patients provided written informed consent.

### Data collection

The clinical information, clinicopathological parameters and preoperative routine blood tests of the patients were collected. Clinical information included patient age, FIGO stage, postoperative therapeutic scheme, smoking history, medical complications and surgical complications. Pathological features mainly included pelvic lymph node (PLN) metastasis status, tumor size, histological grade, tumor invasion depth, lymphovascular invasion and surgical margin. The routine blood tests at 1 week before surgery included those to evaluate WBC, Hb, monocytes, neutrophils, lymphocytes, PC, MPV and the platelet distribution width (PDW), along with NLR, PLR, MLR and MPV/PC calculated based on the abovementioned results. Additionally, the optimal cutoff values of some continuous variables were determined by the receiver operating characteristic (ROC) curve to convert them into categorical variables [12].

### Statistical analysis

The intergroup differences in variable data were analyzed by chi-squared test or t-test. The Kaplan–Meier curve was adopted for survival analysis and tested using the log-rank test. To evaluate the discriminating ability of MPV/PC, NLR, PLR and MLR in cervical cancer prognosis, ROC curves were plotted to preliminarily evaluate the area under the curve (AUC). Next, the prognostic factors for patients were evaluated using the univariate Cox proportional hazard regression model. Later, the multivariate Cox proportional hazard model was constructed using the reverse step-by-step deletion method. Afterward, the variables selected by the multivariate Cox model were used to construct the nomogram. Typically, the C-index and AUC were mainly used to assess the performance of the nomogram. Bootstrap sampling was repeated 1000 times for nomogram verification and evaluation calibration. The rms module of the R programming language, SPSS 22.0 and GraphPad Prism 5 were employed for statistical analysis. All *p* values were two-sided, and a difference of *P* < 0.05 indicated statistical significance.

## Results

### Patient characteristics

In total, 408 cervical cancer patients who had undergone radical surgery were enrolled in this study. The clinicopathological features of patients in the primary cohort (*n* = 283) and validation cohort (*n* = 125) are displayed in Table 1. In the primary cohort, the median patient age was 47 (range, 24–84) years. In the validation group, the median follow-up period was 47 (range, 3–120) months, and the median patient age was 43 (range, 24–67) years. The optimal cut-off value based on the primary cohort was were selected by ROC curve analysis as follows: MPV/PC (MPV/PC ≤ 0.41, MPV/PC > 0.41), NLR (NLR ≤ 2.75, NLR > 2.75), PLR (PLR ≤ 128, PLR > 128) and MLR (MLR ≤ 0.27, MLR > 0.27). The clinicopathological features of patients in the two cohorts were comparable, except for the tumor invasion depth. The correlations of MPV/PC with the clinicopathological features of cervical cancer patients are displayed in Table 2. In the primary cohort, MPV/PC was only negatively correlated with PLR (*P* = 0.007) but not related to other pathological parameters. Similar results were also obtained in the validation cohort.

**Table 1:** Clinicopathological characteristics of patients with cervical cancer in primary cohort and validation cohort

Characteristic	Primary Cohort (*n* = 283)		Validation Cohort (*n* = 125)	
	No. of Patients	%	No. of Patients	%
Age				
≤ 45	136	48.1	62	49.6
> 45	147	51.9	63	50.4
Histological grade				
G1	22	7.8	12	9.2
G2	147	51.9	67	53.6
G3	114	40.3	46	36.8
Tumor invasion depth				
≤ 1/2	219	77.4	72	57.6
> 1/2	64	22.6	53	42.4
Tumor size				
≤ 4	155	54.8	78	60.0
> 4	128	45.2	52	40.0
Lymphovascular invasion				
No	233	82.3	87	69.6
Yes	50	17.7	38	30.4
FIGO stage				
IA	65	23.0	31	24.8
IB	166	58.7	59	47.2
IIA	52	18.4	35	28.0
Radiotherapy				
No	241	85.2	90	72.0
Yes	42	14.8	35	28.0

**Table 2:** Correlations between preoperative SII and clinicopathological characteristics in primary and validation cohort

Clinical parameter	Primary Cohort				Validation Cohort			
	MPV/PC ≤ 0.41 (141)	MPV/PC>0.41 (142)	χ^2^	*P*	MPV/PC ≤ 0.41 (55)	MPV/PC>0.41(70)	χ^2^	*P*
Age			0.09	0.768			1.40	0.237
≤ 45	69	67			24	38		
> 45	72	75			31	32		
Histological grade			0.33	0.850			0.30	0.860
G1	12	10			5	7		
G2	74	73			31	36		
G3	55	59			19	27		
Tumor invasion depth			0.78	0.376			0.01	0.907
≤ 1/2	106	113			32	40		
> 1/2	35	29			23	30		
Tumor size			0.01	0.957			1.48	0.223
≤ 4	77	78			27	42		
> 4	64	64			28	28		
Lymphovascular invasion			0.12	0.734			2.81	0.094
No	115	118			34	53		
Yes	26	24			21	17		
FIGO stage			3.51	0.173			0.58	0.749
IA	39	26			14	17		
IB	78	88			24	35		
IIA	24	28			17	18		
Radiotherapy			2.71	0.099			0.32	0.574
No	125	116			41	49		
Yes	16	26			14	21		
NLR			0.18	0.674			1.73	0.189
NLR ≤ 2.75	77	74			18	31		
NLR > 2.75	64	68			37	39		
PLR			7.26	0.007^*^			17.96	< 0.001^*^
PLR ≤ 128	52	75			21	53		
PLR > 128	89	67			34	17		
MLR			1.36	0.243			0.54	0.462
MLR ≤ 0.27	90	81			28	31		
MLR > 0.27	51	61			27	39		

*MPV/PC* mean platelet volume/platelet count, *NLR* neutrophil lymphocyte ratio, *PLR* platelet lymphocyte ratio, *MLR* monocyte lymphocyte ratio

### Survival analysis

**Fig. 1:**
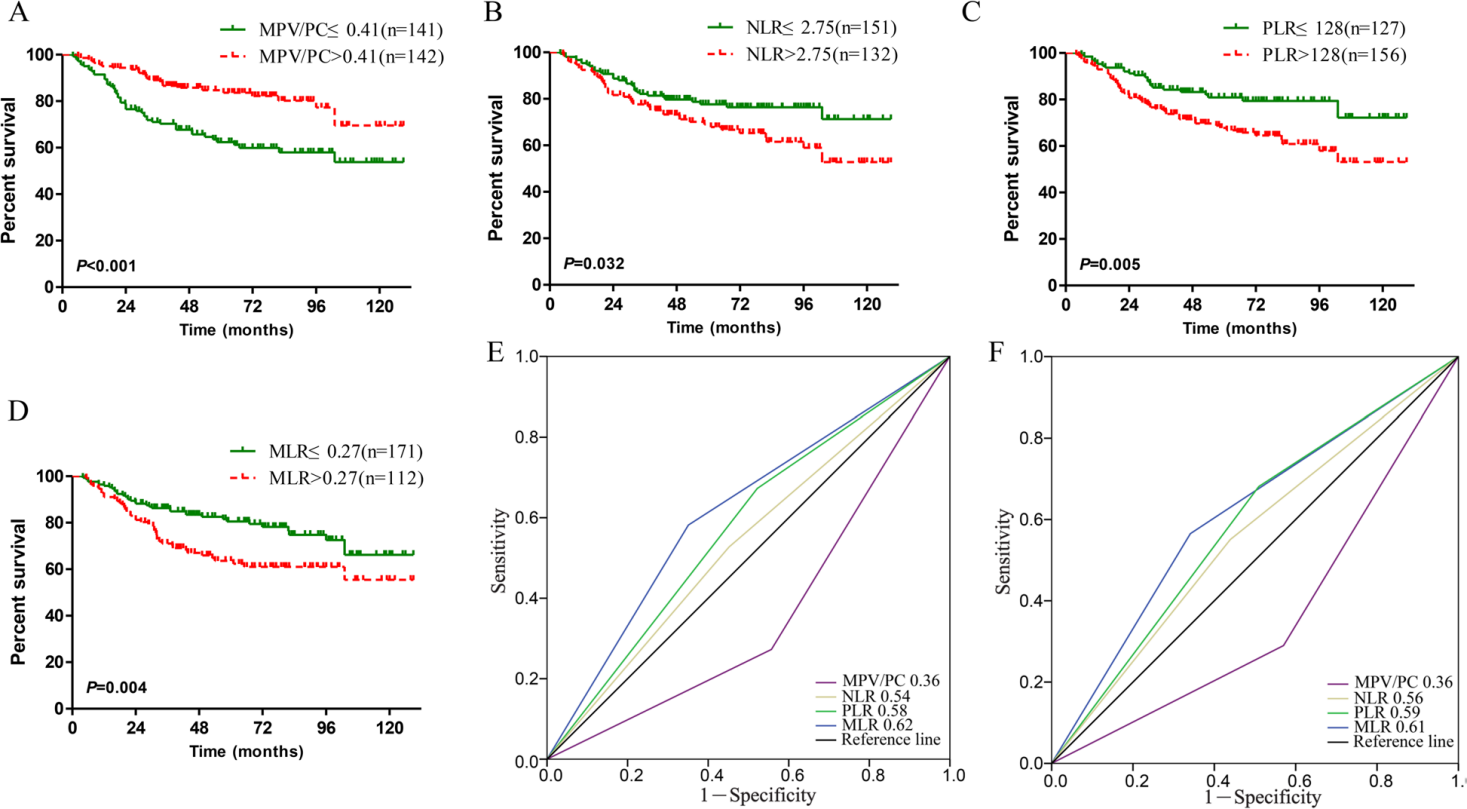
Kaplan–Meier curves for cervical cancer patients stratified by (**A**) MPV/PC, **B** NLR, **C** PLR and **D** MLR in the primary cohort. The predictive ability of MPV/PC in cervical cancer patients was compared with NLR, PLR and MLR using ROC curves at 3 years (**E**) and 5 years (**F**) in the primary cohort

**Fig. 2:**
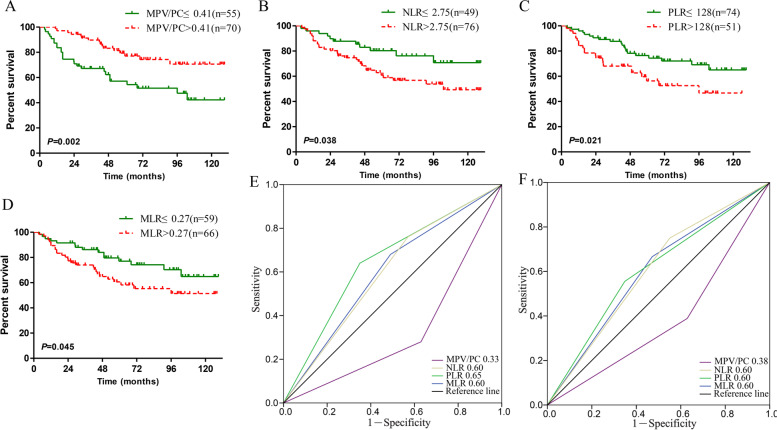
Kaplan–Meier curves for cervical cancer patients stratified by (**A**) MPV/PC, **B** NLR, **C** PLR and **D** MLR in the validation cohort. The predictive ability of MPV/PC in cervical cancer patients was compared with NLR, PLR and MLR using ROC curves at 3 years (**E**) and 5 years (**F**) in the validation cohort

In the primary cohort, the Kaplan–Meier survival curves of the MPV/PC, NLR, PLR and MLR indices are presented in Fig.1A-D. The OS of patients with MPV/PC ≤ 0.41 was significantly lower than that of patients with MPV/PC > 0.41 (*P* < 0.001; Fig. 1A). Additionally, the NLR, PLR and MLR indices may be used to evaluate the prognosis of cervical cancer patients, and the differences were statistically significant (Fig. 1B-D). Subsequently, the prognostic values of the 4 indices were compared using the AUC value. Compared with other systemic inflammatory indices, the MPV/PC index displayed greater AUC values in predicting the 3- and 5-year survival rates for cervical cancer patients, indicating that MPV/PC had better prognostic value than NLR, PLR or MLR for cervical cancer patients (Fig. 1E-F). Additionally, similar results were also obtained in the validation cohort (Fig. 2A-F).

In the primary cohort, the univariate Cox proportional hazard regression model indicated that tumor invasion depth, tumor size, lymphovascular invasion, FIGO stage, MPV/PC, NLR, PLR and MLR were prognostic factors for cervical cancer patients (Table 3). The multivariate Cox proportional hazard regression model indicated that lymphovascular invasion, FIGO stage and MPV/PC were independent prognostic factors for cervical cancer patients (Table 3). In the validation cohort, tumor invasion depth was not significantly related to the prognosis of cervical cancer patients, a finding that might be related to the small sample size. The other results were consistent with those obtained in the primary cohort (Table 4).

**Table 3:** Univariate and multivariate cox regression analyses for overall survival in cervical cancer patients in primary cohort

Variables	Univariate analysis		Multivariate analysis	
	HR (95%CI)	*P* value	HR (95%CI)	*P* value
Age				
≤ 45 years vs. > 45 years	0.99 (0.64–1.55)	0.984		
Histological grade		0.228		
G1	Ref.	–		
G2	1.01 (0.43–2.81)	0.486		
G3	1.60 (0.63–4.05)	0.325		
Tumor invasion depth				
> 1/2 vs. ≤1/2	1.79 (1.11–2.87)	0.017^*^	0.99 (0.57–1.71)	0.970
Tumor size				
> 4 vs. ≤4	2.10 (1.33–3.30)	0.001^*^	1.25 (0.75–2.08)	0.397
Lymphovascular invasion				
Yes vs. No	2.11 (1.30–3.43)	0.003^*^	2.27 (1.38–3.73)	0.001^*^
FIGO stage		< 0.001^*^		< 0.001^*^
IA	Ref.		Ref.	
IB	3.37 (1.44–7.89)	0.005^*^	3.85 (1.60–9.29)	0.003^*^
IIA	8.18 (3.37–19.86)	< 0.001^*^	10.16 (3.69–27.98)	< 0.001^*^
Radiotheropy				
Yes vs. No	0.70 (0.35–1.40)	0.312		
MPV/PC				
> 0.41 vs. ≤0.41	0.42 (0.26–0.67)	< 0.001^*^	0.32 (0.19–0.51)	< 0.001^*^
NLR				
> 2.75 vs. ≤2.75	1.62 (1.04–2.54)	0.034^*^	1.53 (0.97–2.43)	0.070
PLR				
> 128 vs. ≤128	1.96 (1.22–3.15)	0.006^*^	1.57 (0.92–2.68)	0.096
MLR				
> 0.27 vs. ≤0.27	1.83 (1.18–2.85)	0.007^*^	1.50 (0.90–2.51)	0.122

*MPV/PC* mean platelet volume/platelet count, *NLR* neutrophil lymphocyte ratio, *PLR* platelet lymphocyte ratio, *MLR* monocyte lymphocyte ratio

**Table 4:** Univariate and multivariate cox regression analyses for overall survival in cervical cancer patients in validation cohort

Variables	Univariate analysis		Multivariate analysis	
	HR (95%CI)	*P* value	HR (95%CI)	*P* value
Age				
≤ 45 years vs. > 45 years	1.48 (0.80–2.72)	0.212		
Histological grade 0.859				
G1	Ref.	–		
G2	1.27 (0.44–3.68)	0.660		
G3	1.10 (0.365–3.32)	0.865		
Tumor invasion depth				
> 1/2 vs. ≤1/2	1.58 (0.86–2.89)	0.142		
Tumor size				
> 4 vs. ≤4	2.03 (1.10–3.74)	0.024^*^	1.29 (0.67–2.52)	0.449
Lymphovascular invasion				
Yes vs. No	2.20 (1.19–4.07)	0.012^*^	2.63 (1.34–5.13)	0.005^*^
FIGO stage		0.001^*^		< 0.001^*^
IA	Ref.		Ref.	
IB	6.13 (1.42–26.43)	0.015^*^	7.91 (1.79–34.92)	0.006^*^
IIA	13.16 (3.08–56.19)	0.001^*^	20.43 (4.46–93.65)	< 0.001^*^
Radiotheropy				
Yes vs. No	1.42 (0.74–2.71)	0.281		
MPV/PC				
> 0.41 vs. ≤0.41	0.38 (0.21–0.72)	0.003^*^	0.35 (0.18–0.66)	0.001^*^
NLR				
> 2.75 vs. ≤2.75	2.04 (1.03–4.06)	0.042^*^	1.96 (0.98–3.94)	0.058
PLR				
> 128 vs. ≤128	2.02 (1.10–3.71)	0.024^*^	1.74 (0.92–3.31)	0.090
MLR				
> 0.27 vs. ≤0.27	1.89 (1.00–3.55)	0.049^*^	1.57 (0.81–3.04)	0.184

*MPV/PC* mean platelet volume/platelet count, *NLR* neutrophil lymphocyte ratio, *PLR* platelet lymphocyte ratio, *MLR* monocyte lymphocyte ratio

### Construction and validation of the nomogram

**Fig. 3:**
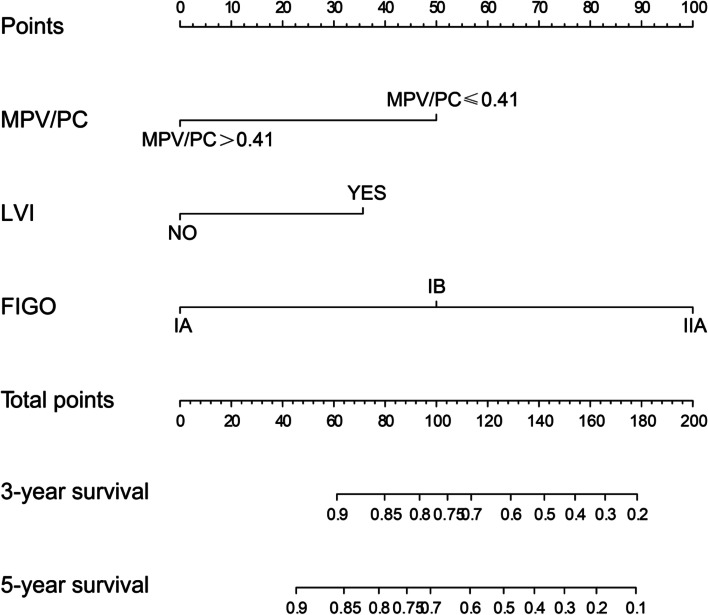
Nomogram based on MPV/PC, LVI and FIGO in cervical cancer

**Fig. 4:**
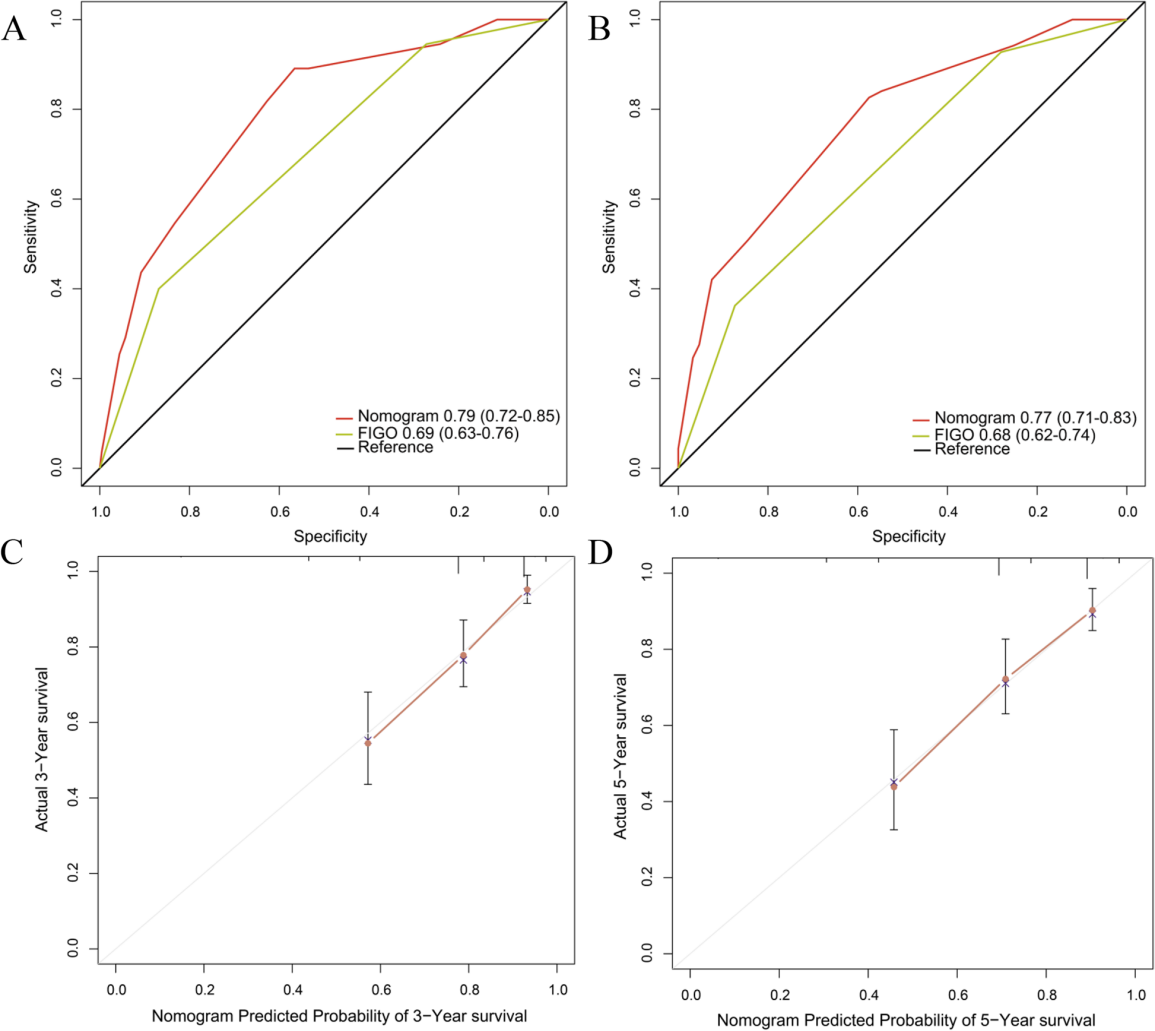
The 3-year survival rate (**A**) and 5-year survival rate (**B**) of cervical cancer patients predicted by the nomogram were highly consistent with the actual observed values in the primary cohort. Ability of the ROC analysis nomogram to predict the 3-year survival rate (**C**) and 5-year survival rate (**D**) of cervical cancer patients. The nomogram had a larger AUC than FIGO staging in the primary cohort

**Fig. 5:**
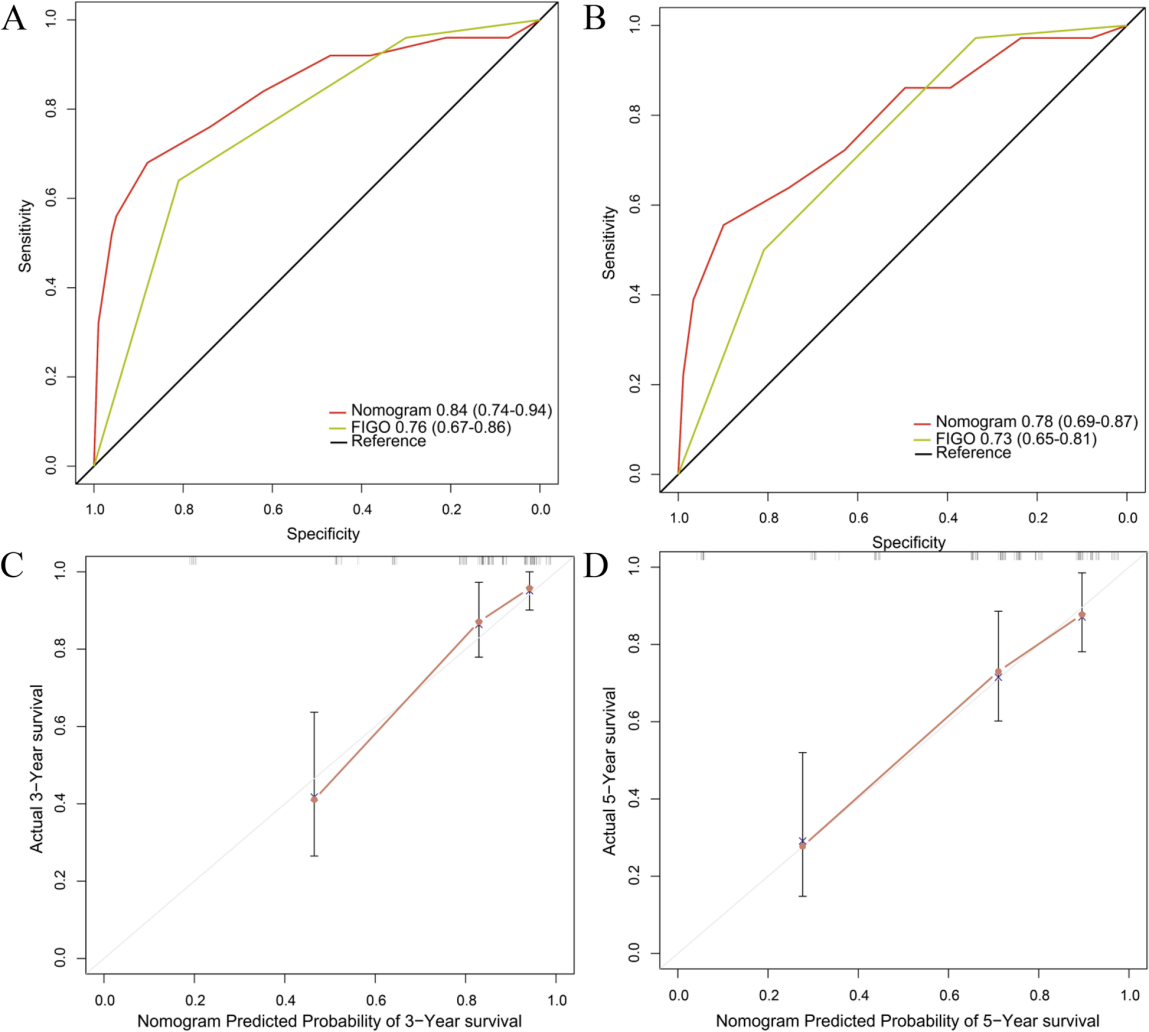
The 3-year survival rate (**A**) and 5-year survival rate (**B**) of cervical cancer patients predicted by the nomogram were highly consistent with the actual observed values in the validation cohort. Ability of the ROC analysis nomogram to predict the 3-year survival rate (**C**) and 5-year survival rate (**D**) of cervical cancer patients. The nomogram had a larger AUC than FIGO staging in the validation cohort

In the primary cohort, independent risk factors for cervical cancer, including MPV/PC, FIGO stage and lymphovascular invasion, were used to construct nomogram models to predict the 3- and 5-year OS of cervical cancer patients (Fig. 3). The C-index of the as-constructed nomogram was 0.77, significantly higher than the 0.68 of the FIGO stage (*P* < 0.001). AUC analysis revealed that the AUC value of the nomogram was significantly greater than that of the FIGO stage (Fig. 4A and B), indicating that the nomogram might be used to assess the prognosis for patients with resectable cervical cancer and that it was more accurate than the traditional FIGO stage. In internal verification, the calibration curve of the model displayed favorable consistency between the predicted and actual values (Fig. 4C-D), demonstrating the extremely reliable repeatability of the nomogram. Furthermore, external verification of the nomogram was conducted using the validation cohort data. The C-index of the nomogram was 0.82, which was significantly higher than the 0.72 of FIGO stage (*P* < 0.001). The AUC value of the nomogram was apparently higher than that of the FIGO classification system (Fig. 5A and B). Additionally, the calibration curve of the nomogram showed good consistency between the predicted 3−/5-year survival rates and actual observed values (Fig. 5C and C). The above results suggested that the nomogram might serve as a tool to predict the survival of patients more effectively and accurately with resectable cervical cancer.

## Discussion

Since scholars first reported the increased platelet count in cancer patients in 1872 [13], the platelet changes in blood from patients with malignant tumors at different sites have been successively investigated. Platelets are one of the bioactive visible blood components produced by mature megakaryocytes in bone marrow and affect the inflammatory response and immune regulation in the body [14, 15]. Platelets extensively exist in the peripheral blood circulation and exert their role in tumor invasion, hematogenous metastasis and distal organ colonization through surface molecules or secreting related factors [16, 17]. PC and MPV are the two most important indices to evaluate platelet function, and their combination in the MPV/PC index has recently been used in the prognosis evaluation of malignant tumor patients. MPV/PC was first used as the prediction index for long-term mortality after myocardial infarction [18]. Recently, Cho et al. revealed the expression level and diagnostic value of MPV/PC in hepatocellular carcinoma (HCC) patients [6]. Subsequently, Gu et al. found that the OS of HCC patients with high MPV/PC was significantly poorer [11]. In lung carcinoma, Inagaki et al. found that MPV was expressed at low levels in lung carcinoma patients, while PC was highly expressed. The OS of non-small cell lung cancer (NSCLC) patients with low MPV/PC was significantly superior to that of patients with high MPV/PC, and MPV/PC was an independent prognostic factor for locally advanced NSCLC [7]. In colorectal cancer (CRC), the low MPV/PC in patients suggests a low TNM stage and less LNM [9]. We confirmed that cervical cancer patients with high MPV/PC had a poor prognosis, and MPV/PC was an independent prognostic factor for cervical cancer patients. The ROC curve revealed that MPV/PC showed higher prognostic value than NLR, PLR and MLR in cervical cancer. Afterward, multivariate analysis identified the clinicopathological variables MPV/PC, FIGO stage and lymphovascular invasion, which were incorporated to construct the nomogram. The nomogram exhibited a high accuracy in predicting survival (C-index = 0.78) and a significantly higher predictive ability in the survival of the primary cohort than the FIGO classification system. These results were then verified by a group of independent external verification cohort data. MPV/PC may serve as a prognostic and therapeutic marker that contributes to the early formulation of a more accurate and timelier individualized therapeutic scheme.

Malignant tumors may result in alterations in platelet parameters, but the mechanism by which MPV/PC can be used to predict the prognosis of malignant tumors has not yet been completely illustrated and may be related to the following factors. Bone marrow hyperplasia is active in malignant tumor patients, tumor cells produce thrombogenic factors, and the body fluid environment concentration that promotes the generation of bone marrow megakaryocytes is elevated in the blood circulation [15]. The number of cytokines that promote tumor growth is increased in tumor patients. Additionally, these cytokines, such as interleukin (IL)-1, IL-3, IL-6, IL-17, IL-18 and tumor necrosis factor-α (TNF-α), specifically stimulate increased production of platelets, [19, 20]. Stone et al. revealed that IL-6 promotes the secretion of more thrombogenic factors by the liver, while ovarian cancer cells secrete IL-6, which causes an increased amount of thrombogenic factors to act on the bone marrow, resulting in increased platelet production and a changed platelet morphology [21]. Furthermore, malignant tumor cells secrete granulocyte macrophage-colony stimulating factors, thus accelerating megakaryocyte production and thrombosis [15]. Malignant tumors consume a large amount of patient energy; thus, tumor patients may develop chronic blood loss, malnutrition and tissue necrosis, which affect platelet morphological parameters [15]. Malignant tumor cells can secrete and stimulate increased platelets to release transforming growth factor (TGF), directly stimulating the growth of certain tumor cells; however, proliferating tumor tissues produce more stimulating factors to accelerate bone marrow megakaryocyte production, thus forming a vicious cycle [15].

This study has certain limitations. (1) This study was a retrospective single-center study, which might be associated with selection bias. (2) Heterogeneity existed in the treatments that patients received after surgical resection, likely affecting different clinical outcomes. (3) The cutoff values in this study were not verified in other cohorts. Thus, more large-scale, multicenter clinical studies are warranted to verify our research results.

## Conclusion

As a noninvasive, lost-cost, simple and repeatable index, MPV/PC is a novel independent prognostic index for patients with resectable cervical cancer. Compared with the traditional FIGO classification system, the nomogram that integrates MPV/PC maybe reliably predict the survival of cervical cancer patients after radical surgery.

## Data Availability

The dataset supporting the conclusions of this article is available at The Second Affiliated Hospital of Soochow University from the corresponding author on reasonable request .
